# Quaternary arrangements of membrane proteins: an aquaporin case

**DOI:** 10.1042/BST20241630

**Published:** 2024-12-13

**Authors:** Maria Hrmova

**Affiliations:** School of Agriculture, Food and Wine, and Waite Research Institute, Faculty of Sciences, Engineering and Technology, University of Adelaide, Glen Osmond, South Australia 5064, Australia

**Keywords:** hetero-and homo-oligomerization, integration and trafficking, lipid bilayers, membrane protein complexes, single-molecule-based, subcellular localization

## Abstract

Integral polytopic α-helical membrane transporters and aquaporins move and distribute various molecules and dispose of or compartmentalize harmful elements that gather in living cells. The view shaped nearly 25 years ago states that integrating these proteins into cellular membranes can be considered a two-stage process, with hydrophobic core folding into α-helices across membranes to form functional entities (Popot and Engelman, 1990; *Biochemistry* **29**, 4031–4037). Since then, a large body of evidence cemented the roles of structural properties of membrane proteins and bilayer solvent components in forming functional assemblies. This mini-review updates our understanding of multifaced factors, which underlie transporters integration and oligomerization, focusing on water-permeating aquaporins. This work also elaborates on how individual monomers of bacterial and mammalian aquaporin tetramers, interact with each other, and how tetramers form contacts with lipids after being embedded in lipid bilayers of known composition, which mimics bacterial and mammalian membranes. Although this mini-review describes findings acquired using current methods, the view is open to how to extend this knowledge through, e.g. single-molecule-based and *in situ* cryogenic-electron tomography techniques. These and other methods could unravel the sources of entropy for membrane protein assemblies and pathways underlying integration, folding, oligomerization and quaternary structure formation with binding partners. We could expect that these exceedingly interdisciplinary approaches will form the basis for creating optimized transport systems, which could inspire bioengineering to develop a sustainable and healthy society.

## Introduction

It has been nearly 25 years since the hypothesis was put forward that incorporating most integral (or intrinsic) polytopic α-helical membrane proteins (spanning membrane and classified in Types-I to Types-VI) in cellular membranes can be considered a two-stage process. During stage I, hydrophobic regions of membrane proteins fold into α-helices crossing a lipid bilayer to give rise to functional proteins during stage II [1,2]. Since then, a large body of evidence has accumulated to specify the roles of structural properties of membrane proteins and lipid bilayers, where membrane proteins fold and form functional assemblies. Biophysical studies such as cryogenic-electron microscopy (cryo-EM) combined with 3D reconstruction were instrumental in defining the interactions between transmembrane protein α-helices and lipid bilayers [3]. These efforts contributed to formulating the unifying models for α-helical protein biogenesis, which emphasize the contributions of lipids, termed finely-tuned molecular machines [4–7] in membrane protein folding and oligomerization. For example, in human aquaporin 1 [AQP1; Protein Data Bank (PDB) accession 1H6I], a polar tripartite motif at transmembrane α-helices 2 and 5 was a key to directing its α-helical packing, topogenesis, monomer folding and tetramerization. This mechanism carved by evolutionary divergence could have general importance [8].

This mini-review is not exhaustive and focuses on the last three years of progress with earlier concepts contextually mentioned.

## Experimental approaches to investigations of membrane proteins in lipid bilayers

The approaches describing biochemical and biophysical properties of α-helical membrane proteins have significantly expanded since the formulation of the two-stage hypothesis [1,2]. For example, more recent methods to monitor protein binding in membranes include switchSENSE® technology [9], while cell imaging, cryo-EM, and *in situ* cryogenic-electron tomography (cryo-ET) visualize macromolecules *in situ* [10]. However, cryo-ET must improve interpretability or assignment identity with diminish low-resolution and noise problems via template-free or template-based setups [11]. An example of cryo-ET achievement is a near-complete atomic model of a human endoplasmic reticulum (ER)-bound translocon [12]. Other techniques of studying membrane proteins include synthetic chemistry combined with nanodevices [13] and model liposomes linked with microfluidics sizing-based assays, circular dichroism, fluorescence correlation spectroscopy, thermodynamic quantifications and mechanistic approaches to protein embedding in lipid bilayers [14]. These methods could be combined with high-precision single-molecule total internal reflection fluorescence microscopy to evaluate, amongst other properties, distances and dynamics between membrane oligomers in bilayers of live cells [15,16]. Additional approaches include pulsed interleaved excitation fluorescence cross-correlation spectroscopy, which examines the oligomerization of membrane proteins in live cells and measures single-cell-based protein expression levels and diffusion coefficients [17], and single-molecule-based tracking exploring the dynamics of protein movements [18]. These methods could be supplemented by techniques that evaluate scattering length density profiles of bilayers via neutron and X-ray diffraction to determine the location and orientation of molecules between asymmetric membrane leaflets [19].

Theoretical and computational approaches also contributed to descriptions of membrane proteins and nuanced mechanisms of bilayer asymmetry that govern structural and dynamic coupling between compositionally distinct bilayer leaflets [20]. The definitions of force-field parameters [21] allowed describing properties of homogeneous bilayers in bacterial, mammalian and cancer cells (area, volume, and thickness properties) [22] and mechanical properties of solid-ordered bilayers (area per lipid, density vesicle profiles, bending rigidity coefficient, area compressibility properties) via molecular dynamics (MD) simulations [23]. The latest developments include the coarse-grained Martini 3 OliGo̅mers method, developed for large-scale simulations, to realistically describe higher-order biomolecules while considering folding and unfolding events [24]. The Martini 3 OliGo̅mers method was applied to the assembly/disassembly of tetrameric bovine AQP1 (PDB accession 1J4N), revealing that it formed an exceptionally stable tetrameric structure. It also suggested that stability was due to a mismatch between the AQP1 hydrophobic regions and the bilayer thickness [24].

To assist these methods, structural repositories provide a wealth of information: specifically, the PDB [25] with >227 000 entries and nearly 1.1 million computed models (as of November 15, 2024), combined with the Cambridge Structural Database, enlisting >1.25 million small-molecule structures [26]. Other valuable resources are 2024 Nobel Prize-awarded AlphaFold [27,28] and RoseTTAFold [29,30] combined with AlphaFill [31], AlphaFold-Multimer [32], and Cosmic^2^ [33], which integrate structural analyses with machine learning.

## Integration of membrane proteins in lipid bilayers and oligomerization

Some integral polytopic α-helical membrane proteins form ordered oligomeric quaternary assemblies, which underlie their function with profound physiological consequences [34,35]. Although a membrane protein monomer could sometimes function, oligomeric structures offer advantages. Often, in lipid bilayers, the occurrence of mono- and oligomeric membrane proteins could take place simultaneously, as shown with AQPZ (PDB accession 1RC2) using high-speed atomic force microscopy (HS-AFM) and kinetic membrane elastic theory, which quantify membrane-mediated interactions at high resolution and define elastic properties in the bilayers [36].

The two major features that underscore the integration and formation of α-helical membrane protein quaternary assemblies are properties of: (i), membrane proteins, such as interaction patterns and topology, which confine and underly the orientation of protomers to facilitate assembly [37]; and (ii), a lipid bilayer-forming solvent, which potentiates folding of membrane proteins in a native environment [38,39]. Factors that affect membrane transport protein integration and oligomerization can be subdivided into those of 3D structural properties, the presence of GXXXG and leucine heptad sequence motifs, properties of loops facing extracellular and intracellular environments, N- and C-terminal termini modifications, lipid release potential, structural stability, quaternary contacts, cooperativity between protomers, transport activity, and hydrophobic matching within a lipid bilayer, while those of bilayer properties include lateral pressure, lipid composition and chemistry, and the thickness of a bilayer core [35,36,39–42]. Other factors that play roles in these processes are post-translational modifications such as e.g. phosphorylation and lipidations (discussed below in detail), and changes through interactions with ‘proteomimic’. The latter term involves interactions with soluble or membrane proteins, peptides, peptide mimetics and small molecule ligands, which could enhance, modify or block protein function; this could be exploited in drug and herbicide design [39]. In other transporters, such as solute carrier six family members, a single-molecule fluorescence microscopy revealed that they form a range of monomeric to multimeric assemblies with complex stoichiometries, where all transmembrane α-helices contributed to oligomer interfaces, except a bundle domain [42].

Membrane proteins interact with annular lipids localized in their immediate vicinity, which restricts membrane proteins’ mobility, with an overall entropy increase, and even so much more after membrane proteins form higher-order quaternary structures when the lipid-membrane-protein surface areas are progressively lowered [35]. In the latter case, only obligate oligomeric membrane proteins are functional. Conversely, facultative membrane proteins may operate as monomers or oligomers — such is the case of AQPs [35,36,41], although this distinction is not always clear-cut.

Other functions that underscore membrane protein integration and organization in bilayers are the roles of lipid-binding modules (e.g. 150 amino acid residue discoidin-type C2 or 30-residue long basic-rich domains occurring in yeasts, mammals and plants) and protein lipidation, which targets proteins to intracellular membranes allowing them to operate spatially at defined cellular locations [43]. Specifically, in *Saccharomyces cerevisiae* plasma membranes monitoring with fluorescent probes-tagged lipid-binding domains in real-time visualized lipid species and how they associate with cellular membranes. This approach helped to understand the signalling roles of protein complexes in plasma membranes after being targeted to plasma membranes. Similarly, co- and post-translational prenylation (at C-termini of Cys residues), myristoylation (at N-termini of Gly residues), palmitoylation (additions to Cys residues along protein chains), and O- and N-acylation (Ser and Lys residues) could increase membrane attachments through a higher hydrophobicity [43]. These adjustments target co- and post-translationally modified membrane proteins to their locations and combined with engineered membrane proteins, it is possible to clarify their exact function in plasma membranes.

## Biosynthesis and trafficking of membrane proteins

Integral polytopic α-helical membrane proteins are incorporated into ER membranes after they are synthesized by ribosomes, integrated into ribosome-associated ER membranes and trafficked via the Golgi system to their target membrane locations through elaborate trafficking machinery [19,42,44–46]. This machinery in eukaryotic cells includes SEC24C or SEC24D protein transport proteins, a Coat Protein Complex I (COPI) and a heptameric Coat Protein Complex II (COPII), which traffics membrane proteins in cells through COPII-vesicular machinery [45]. It is also significant that plasma membrane curvature regulates lipid homeostasis and contacts with ER membranes, which stabilize the inserted membrane protein oligomers [47]. This could affect their post-translational modifications, sorting, and trafficking [36]. During insertion and trafficking, the synthesized membrane proteins must be correctly folded, including the packing of their C-terminal α-helices facing ER membranes. It has been suggested that protein components of SEC24 might sense the folding of transporters or recognize their conformational equilibrium rather than participate in oligomerization [42,44]. Although, these events may not be neatly separated and could coincide [48]. To clarify these aspects, new revolutionary approaches, such as single-molecule atomic force microscopy (AFM) and single-molecule-forced unfolding using magnetic tweezers, could unravel forces [hydrogen bonds (H-bonds), hydrophobic and electrostatic interactions, van der Waals forces] as the source of entropy for folding and oligomerization of membrane proteins [48].

## Quaternary structure formation of aquaporins

Several principles of AQPs homo- or heteromeric quaternary structure formation were described, and exact determinants differ in each case. Dynamic imaging resolved the association/dissociation processes of AQP monomers [24,36], providing evidence that the mismatch between proteins’ hydrophobic regions and membrane thickness could drive membrane protein oligomerization. In AQPs, at the structural levels, several other factors could favour oligomerization, such as the chemical nature of lipids and lipidic environments where AQPs reside, lipid release during hydrophobic mismatch, quaternary contacts of monomers, structural and proteolytic stability of monomers, which could lead to forming lipid-binding cavities. It would be crucial to reveal how these cavities could affect AQP oligomerization and gating and if their formation is linked to changes in the distributions of secondary structural elements. These factors could modulate AQP function and cooperativity amongst monomers and yield new activity with or without beneficial features [35].

AQPs could be monomerized by even a single mutation, as shown in *E. coli* glycerol facilitator GlpF (PDB accession 1LDA), where polar glutamate was replaced by the apolar Ala residue. In GlpF, four Gln residues co-ordinated a single Mg^2+^ ion in the pore, while a neighbouring Mg^2+^ ion was co-ordinated by four Trp residues; it is unknown if either coordination has functional consequences. This single glutamate to alanine mutation weakened the stability and ability of the GlpF oligomer to form a tetramer, meaning that tetramerization stabilized its quaternary structure. Monomerized *versus* tetramerized forms of GlpF, where the tetramer oligomerized through dimerization, exhibited differences in permeation, and the monomer failed to facilitate polyalcohol flux across the *E. coli* cytoplasmic membranes [49].

However, further, studies of bacterial and human Major Intrinsic Protein families, permeating water and other solutes, indicated that AQPs could not oligomerise upon a single (as detailed above) or several mutations. Here, the exact structural descriptors, such as loop E in human GlpF (PDB accession 1FX8) were responsible for homo-tetramerization [50,51]. Meanwhile, in human AQP2 (PDB accession 4NEF), glutamate 258, residing on a C-terminal cytoplasmic loop, was found to be critical, which upon expression in *Xenopus* oocytes of wild-type or targeted mutants, formed monomeric (mutated into cysteine) or homo-tetrameric (mutated into lysine and wild-type) structures [50]. But, upon co-expression of AQP2 versions, the AQP2-lysine mutant, but not the AQP2-cysteine mutant hetero-tetramerized with wild-type; these tetramers localized to respective ER and the Golgi complex. This inhibition of a correct routing of the hetero-tetrameric AQP2-lysine mutant from the Golgi complex to the plasma membrane exemplified that an impaired routing rather than an impaired function of a mutant was the cause of the nephrogenic diabetes insipidus disease in humans [52].

Several cases of homo- or hetero-oligomerization of AQP quaternary structures (which could be evolutionarily conserved) from Arabidopsis, rice, maize, tobacco, grape and spinach were described upon expression in plant cells or *Xenopus* oocytes. The focus has been on Plasma Membrane Intrinsic Proteins (PIP) isoforms (e.g. PIP1 and PIP2 families) [53–57], although the question remained open if homo- or hetero-tetramers had altered selectivity under normal or adverse environmental settings [58].

For example, hetero-tetramerization of OsPIP1;3 with OsPIP2;2 or OsPIP2;4, when expressed in root cells indicated increased water permeability, while that with OsPIP2;3 did not have the same effect [59]. This effect was assigned to the trafficking of hetero-tetramers to a plasma membrane. In *Beta vulgaris* PIP AQPs, only the *Bv*PIP2;2 isoform formed functional hetero-tetramers with *Bv*PIP1;1 (important for regulation and fine-tuning of water transport), while *Bv*PIP2;1 failed to homo-tetramerize with either *Bv*PIP isoform upon expression in *Xenopus* oocytes [60]. Notably, when analysed by confocal fluorescent microscopy, *Bv*PIP2;1 could not translocate fluorescently labelled BvPIP1;1 from the intracellular to a plasma membrane environment when co-expressed, contrary to *Bv*PIP2;2. Based on the MD simulations and mutational studies, it was concluded that the failure of *Bv*PIP2;1 to hetero-tetramerize was in sequence variations of extracellular loop A. In *Bv*PIP2;1, this loop A folded differently and failed to facilitate inter-chain hydrophobic contacts with other *Bv*PIP isoforms, which hampered tetramer formation [60]. It would be of interest to compare the selectivity of *Bv*PIP homo- and hetero-tetramers and if they permeate other molecules besides water. It was also shown that the *Beta vulgaris* PIP2-PIP1 hetero-tetramers, upon expression in *Xenopus* oocytes, assembled in various stoichiometries (3:1, 1:3, 2:2) depending on cell expression levels [61]. Given that in *Bv*PIP2;1, loop A did not form inter-chain associations with other *Bv*PIP isoforms, and in spinach PIP2;1 loop D, controlled gating via pore occlusion [62], it would be vital to examine how loop movements outside the hydrophobic cores of AQPs affect gating and if this is linked to oligomerization.

Another study of plant homo- and hetero-tetramerization included maize PIP1 and PIP2 AQP isoforms, exhibiting different water-channel activities upon expression in *Xenopus* oocytes [63]. When maize PIP1;2 and PIP2;5 were co-expressed, they were assembled as homo- and hetero-dimers. Structural assessments and analyses of mutant versions AQPs assigned predominantly positively charged, neutral and hydrophobic determinants for α-helical cores of PIP1;2 (lysine, glutamine, Trp and Phe residues) and PIP2;5 (Leu, Phe, Trp and Tyr residues). These side-chains via hydrophobic, aromatic–aromatic, aromatic–sulfur, cation–pi, and H-bonds in the α-helical regions of AQPs were critical for a hetero-oligomeric quaternary assembly. Using microscopy, the fluorescently labelled wild-type and mutant PIP1;2 AQPs co-localized in ER membranes, while after co-expression with PIP2;5, the PIP1;2-PIP2;5 hetero-oligomer localized in a plasma membrane, suggesting strong complementary interactions between the two isoforms.

## Bacterial and human AQP tetramers embedded in lipid bilayers interact with lipids through multiple contacts to help anchoring in bilayers

To analyse how homo-tetrameric bacterial *E. coli* AQPZ (PDB accession 2ABM) [64] and human AQP5 (PDB accession 3D9S) [65], both solved by X-ray crystallography, form contacts and interact with lipids in bilayers of known composition, we conducted MD simulations [22] of tetramers embedded in symmetric bilayers mimicking bacterial or mammalian membranes.

**Figure 1.**
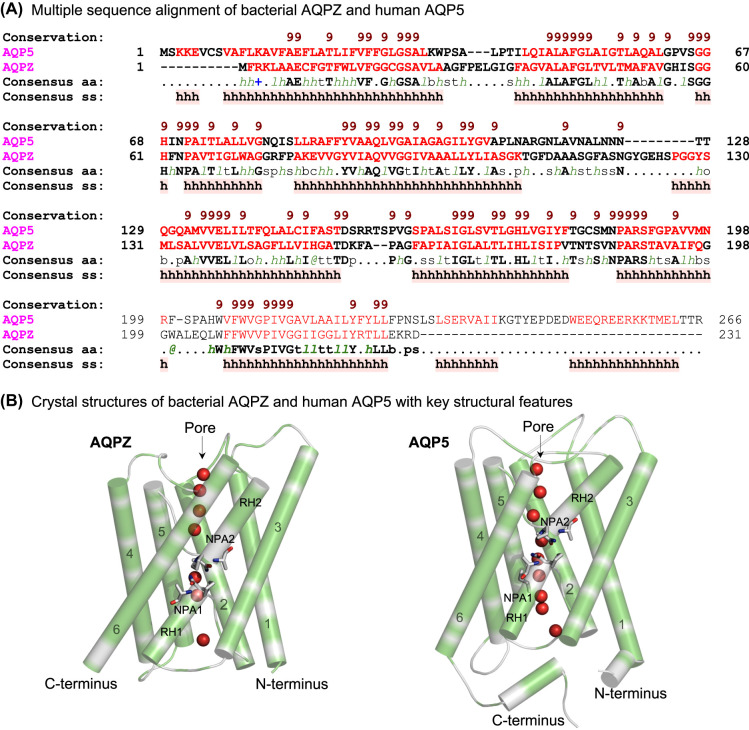
Multiple sequence alignment of *E. coli* AQPZ and human AQP5, and their monomeric structures. (**A**) Sequences were aligned in ProMals3D [66], with the alignment indicating the conservation of residues (absolutely conserved residues on a scale of 5–9; brown). Respective indices ‘s’, ‘p’, ‘l’ and ‘h’ indicate small (A, G, C, S, V, N, D, T, P), polar (D, E, H, K, N, Q, R, S, T), aliphatic (I, V, L) and hydrophobic (W, F, Y, M, L, I, V, A, G) residues. Consensus amino acid residues (Consensus aa) and secondary structure elements (Consensus ss) are shown in two diversified AQPs (magenta). (**B**) Crystal structures of monomeric AQPZ (left) and AQP5 (right) (chains A) indicate dispositions and hydrophobic properties (Ala, Gly, Val, Ile, Leu, Phe, Met, and excluding Trp and Tyr residues; in green) of α-helices 1–6, and projections of re-entrant α-helices RH1 and RH2, and NPA1-NPA2 motifs (cpk sticks), with water molecules (red spheres) located in pores.

Multiple sequence alignment of AQPZ and AQP5 (Figure 1A) and their monomeric structures (chains A) (Figure 1B) indicated that these AQPs have similar dispositions of α-helices 1–6, and re-entrant α-helices 1–2, and NPA1-NPA2 motifs (cpk sticks); the latter two are involved in water and other solute permeation. However, the hydrophobic properties of α-helices 1–6, representing Ala, Gly, Val, Ile, Leu, Phe and Met residues (Figure 1B; in green), differed between the two AQPs, pre-empting that their lipid binding properties might also vary.

### Protomer-protomer contacts in AQPs

**Figure 2.**
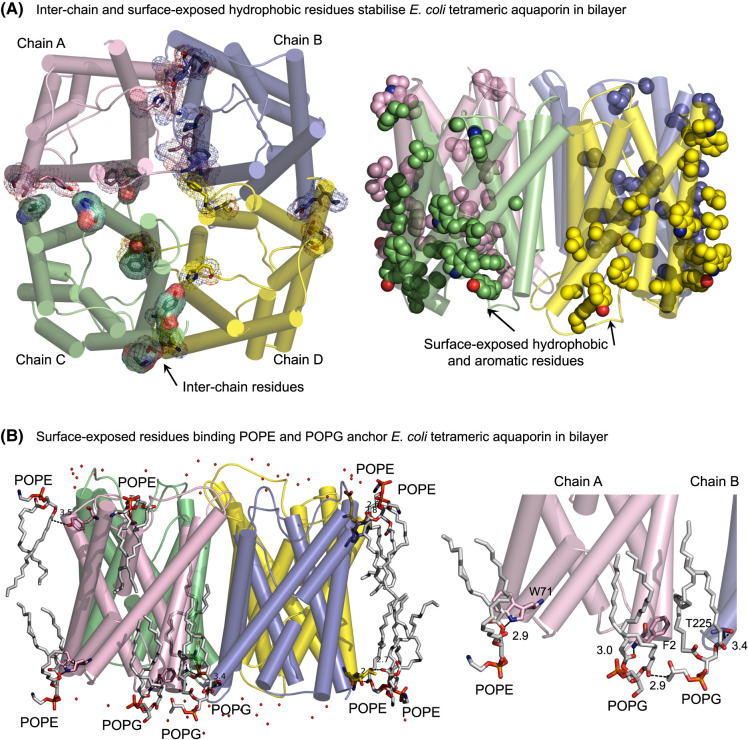
Tetrameric structure of *E. coli* AQPZ reflects the composition of a lipid bilayer, mimicking the bacterial membrane. (**A**) *Left:* Inter-chain residues of AQPZ [64] viewed from the intracellular side down the crystallographic 4-fold symmetry axis, analysed by PDBePISA [67]. Chains A–D are coloured in respective pink, blue, green and yellow, and interacting residues (sticks) between protomers are highlighted in mesh. *Right:* Surface-exposed hydrophobic and aromatic residues (spheres) of tetrameric AQPZ viewed from the membrane plane. (**B**) *Left*: Surface-exposed residues (sticks) of AQPZ, interacting with polar lipid heads in both bilayers at separations between 2.4 and 3.7 Å (dashed lines) viewed from the membrane plane. The image depicts lipids (sticks), protein (cartoon and sticks) and water molecules (non-bonded spheres). *Right*: Details of POPG and POPE lipids contacting residues (sticks) in chains A and B. Separations between 2.4 and 3.4 Å are indicated. Tetramer was embedded in a symmetric bilayer consisting of POPE and POPG lipids simulating the composition of a bacterial membrane [22], using CHARMM-GUI [68,69] and simulated in YASARA [70,71].

**Figure 3.**
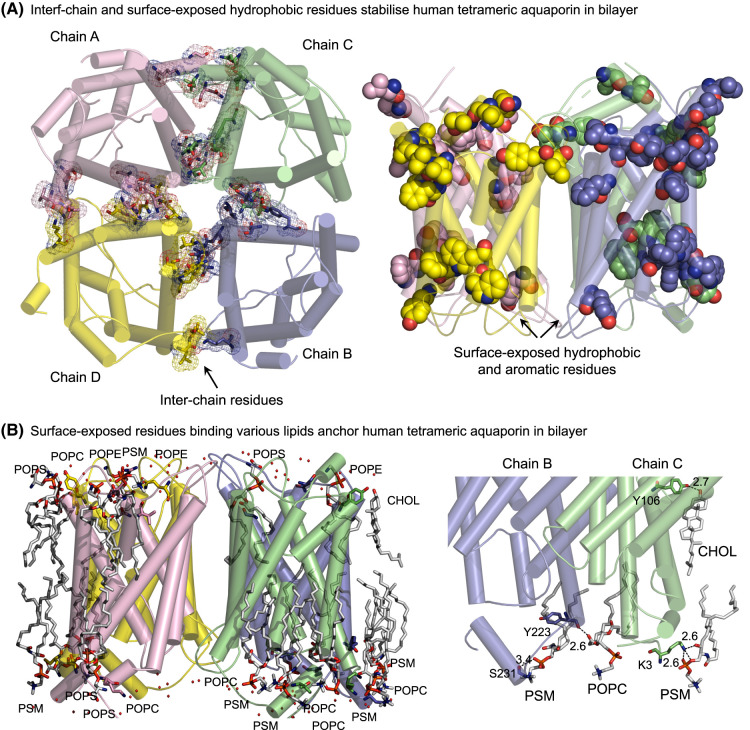
Tetrameric structure of human AQP5 reflects the composition of a lipid bilayer, mimicking the mammalian membrane. (**A**) *Left*: Inter-chain residues of AQP5 [65] viewed from the intracellular side down the crystallographic 4-fold symmetry axis, analysed by PDBePISA [66]. Chains A–D are coloured in respective pink, blue, green and yellow, and interacting residues (sticks) between protomers are highlighted in mesh. *Right:* Surface-exposed hydrophobic and aromatic residues (spheres) of tetrameric AQP5 viewed from the membrane plane. (**B**) *Left*: Surface-exposed residues (sticks) of AQP5 interact with polar lipid heads in both bilayers at separations between 2.5 and 3.7 Å (dashed lines), as viewed from the membrane plane. The image depicts lipids (sticks), protein (cartoon and sticks) and water molecules (non-bonded spheres). *Right*: Details of Chol, PMS, and POPC contacting residues (sticks) in chains B and C, with separations between 2.6 and 3.4 Å indicated. Tetramer was embedded in the symmetric bilayer consisting of CHOL, POPE, POPC, PSM and POPS, simulating the composition of a mammalian membrane [22], using CHARMM-GUI [68,69] and simulated in YASARA [70,71].

Analyses of monomeric interactions of homo-tetrameric *E. coli* AQPZ and human AQP5, using PDBePISA [67] indicated that these contacts were mediated through 30 and 42 residues, respectively (Figures 2A and 3A, left panels). In both AQPs, an equal number of hydrophobic residues (18), were exposed to the neighbouring protomers in A-chains, nine of which were Phe, Tyr and Trp side-chains. From these residues, Phe side-chains dominated with five and six in bacterial and human AQPs, respectively (Figures 2A and 3A, right panels).

### AQPs embedded in lipid bilayers of known compositions

The tetrameric *E. coli* AQPZ was embedded in a symmetric bilayer mimicking a bacterial membrane [22] [POPE 1-palmitoyl-2-oleoyl-*sn*-glycero-3-phosphoethanolamine):POPG (1-palmitoyl-2-oleoyl-*sn*-glycero-3-phosphoglycerol) = 2:1 ratio]. Human tetrameric AQP5 was embedded in a symmetric bilayer mimicking a mammalian membrane [22] [CHOL (cholesterol):POPE:POPC (1-palmitoyl-2-oleoylphosphatidylcholine):POPS (1-hexadecanoyl-2-(9Z-octadecenoyl)-*sn*-glycero-3-phosphoserine):PSM (N-palmitoyl-d-sphingomyelin) = 3.1:1.9:1.6:1.5:1 ratio]. In both cases, we used the Chemistry at Harvard Macromolecular Mechanics Graphical-User-Interface (CHARMM-GUI) for embedding [68,69]. Tetrameric complexes were minimized in YASARA [70] with knowledge-based Yasara2 forcefield (bond distances, planarity of peptide bonds, bond angles, Coulomb terms, dihedral angles, van der Waals forces) combined with the particle-mesh-Ewald energy function for long-range electrostatics at a cut-off 8.0 Å to obtain smoothed electrostatic potentials [71]. Incorrect covalent geometry and conformational stress were removed by short steepest descent minimization (time 5000 fs, 1 ft time steps, 298 K) followed by simulated annealing with 1 fs time step, atom velocities scaled down by 0.9 every 10th step, until convergence at 710 steps with energy improvement of >0.05 kJ/mol per atom during 200 steps. The absence of plant tetrameric AQP co-ordinates and membrane force-field parameters disallowed similar analyses of plant AQPs. Structural images were generated in PyMOL Molecular Graphics System v3.0.7.3 (Schrődinger LLC).

After embedding bacterial and human AQPs in lipid bilayers, on average around 18 and 17 respective lipid-exposed residues were identified in each protomer (Figures 2B and 3B, left panels). Given the dissimilar compositions of bacterial and human membrane bilayers [22], these interactions reflected differences in anchoring AQPs. Tetrameric *E. coli* AQPZ made close contacts with up to 10 POPE (zwitterionic) and POPG (anionic) lipid molecules through the hydrophobic tail regions at the periphery of each homo-tetramer (Figure 2B). Interactions between *E. coli* AQPZ and lipids were also mediated via electrostatic forces, at the separations of 2.6–3.7 Å, e.g. between a positively charged Lys residue and a phosphate group in the hydrophobic head region of POPG. These interactions were also formed via H-bonds between the Arg, Glu, Gln, Tyr and Thr residues and the oxygen atoms of POPG and POPE, containing unsaturated palmitoyl and oleoyl (POPE and POPG; both C16.1/C18:1) fatty acids attached to respective phosphatidylglycerol or phosphatidylethanolamine backbones (Figure 2B). An example of how AQPZ (chains A and B) interacted with POPE and POPG lipid molecules illustrates that Trp, Phe and Thr residues formed contacts with polar head regions, while hydrophobic tails of lipids aligned with hydrophobic surfaces of α-helices (Figure 2B; right panel).

We also revealed molecular interactions with lipids in human AQP5 embedded in the lipid bilayer composed of five types of lipids (Figure 3B). Here, all five lipids: CHOL (neutral), POPE (zwitterionic), POPC (zwitterionic), POPS (anionic), and PSM (anionic) containing unsaturated palmitoyl and oleoyl (POPE; C16.1/C18:1), palmitoyl and oleoyl (POPC; C16.0/C18:1), palmitoyl and oleoyl (POPS; C16.0/C18:1 and phospho-l-serine) and palmitoyl and oleoyl (PSM; C16:0/18:1) fatty acids, made contacts with human AQP5. These lipids participated in aliphatic, aromatic, electrostatic and H-bond interactions with AQP5, with the latter two types of interactions represented by 20 short contacts at the separations between 2.5 and 3.7 Å (Figure 3B). Amongst these contacts, Arg and Lys residues interacted with phosphate headgroups of POPE, POPC, POPS and PSM, and OH groups of CHOL, and their hydrophobic tails were wedged between α-helices. In the latter case, the Tyr106 side-chain formed an H-bond with the CHOL head group at the separation of 2.7 Å, while its hydrocarbon tail aligned with one of the α-helices of AQP5 (Figure 3B; left and right panels).

Our computational analyses of bacterial AQPZ [64] embedded in lipid bilayers mimicking bacterial membranes [22] relate to the experimental findings of *E. coli* aquaglyceroporin GlpF with low permeation activity in liposomes made of native *E. coli* lipids. This finding was, however, surprising, as GlpF should work optimally in its natural membrane environment [72]. In this experimental setup, GlpF permeation was strongly affected by negatively charged lipids, irrespective of the chemical nature of lipid headgroups, and was insensitive to a lateral bilayer pressure. These data imply that GlpF could be modulated by a negative charge density.

Furthermore, *E. coli* AQPZ interaction energies were quantified as a function of a bilayer thickness [36], when embedded in a bilayer of [1,2-dioleoyl-sn-glycero-3-phosphoethanolamine (DOPE), 1,2-dioleoyl-sn-glycero-3-phospho-l-serine (DOPS) and 1,2-dioleoyl-sn-glycero-3-phosphocholine (DOPC) at the ratio 8:1:1], using HS-AFM and kinetic membrane elastic theory. This work revealed that oligomerization was more favourable in lipids matching the protein hydrophobic thickness, whilst interactions with multiple neighbours during protein array formation were favourable in bilayers with a great mismatch. Meanwhile, the archetypal *E. coli* AQPZ interactions with negatively charged phospholipids were conserved between positively charged residues and stabilized its quaternary tetrameric assembly [41,73].

Finally, the computational analyses of dimyristoyl-glycero-phosphocholine (DMPC) bilayer properties with AQP0 (PDB accession 3M9I) indicated that the AQP0 surfaces could induce specific fluid- and gel-phase prone areas [74] and that these regions could influence its mobility. These AQP0-lipid sorting interactions were compatible with a square array oligomerization of tetrameric lens-specific AQP0 (PDB accession 2B6O) encircled by DMPC lipids [75]. This type of oligomerization was recently confirmed for the same AQP0 forming square arrays in lens membranes enriched in sphingomyelin and cholesterol [76]. The latter study of AQP0 in a closed pore conformation solved by cryo-EM in double-layered two-dimensional crystals showed up to seven DMPC molecules with each monomer, forming contacts via Tyr and Trp side-chains.

## Perspectives

- Importance in the field: Structural, biophysical, biochemical and *in vivo* studies emphasized the fundamental importance of mechanistic knowledge for our understanding of quaternary oligomeric arrangements and thermodynamics of membrane transport proteins assemblies and AQPs to understanding their *modus-operandi*.
- Summary of current thinking: A large body of evidence has cemented the roles of structural properties of membrane proteins and lipid bilayers in forming functional membrane proteins. Although this work describes mechanisms using current approaches, the view is open on how future methods could stimulate progress, including interpretability constraints, in emerging single-molecule-based and cryo-ET methods. These improvements will become critical to unravelling the source of entropy of membrane proteins and pathways underlying their integration, folding, oligomerization and quaternary structure formation with binding partners.
- Future directions: Future exceedingly interdisciplinary approaches will form the basis for creating transport proteins and AQPs with optimized features to inspire bioengineering and develop a sustainable and healthy society. We need to resolve and develop: (i), How are homo- and hetero-oligomeric AQP assemblies formed at atomic levels; (ii), Do post-translational modifications contribute/regulate these processes and what is their importance in forming functional proteins in any given biological context; (iii), What are the structural determinants that play roles in the selectivity function of AQP tetramers under normal and a variety of environmental conditions; (iv), For predictions of conformational dynamics of plant AQP tetramers in bilayers, the force-field parameters of plant lipids need to be defined.

## Data Availability

Data contained in the article are available within the submitted material and Figures 1–3.

## Open Access

Open access for this article was enabled by the participation of University of Adelaide in an all-inclusive *Read & Publish* agreement with Portland Press and the Biochemical Society under a transformative agreement with CAUL.

## Abbreviations

3D: three-dimensional
AFM: atomic force microscopy
AQP: aquaporin
CHARMM-GUI: Chemistry at Harvard Macromolecular Mechanics Graphical-User-Interface
CHOL: cholesterol
COPI: Coat Protein Complex I
COPII: Coat Protein Complex II
cryo-EM: cryogenic-electron microscopy
cryo-ET: cryogenic-electron tomography
DMPC: dimyristoyl-glycero-phosphocholine
DOPC: (1,2-dioleoyl-sn-glycero-3-phosphocholine (1-palmitoyl-2-oleoylphosphatidylcholine)
DOPE: 1,2-dioleoyl-sn-glycero-3-phosphoethanolamine
DOPS: 1,2-dioleoyl-sn-glycero-3-phospho-l-serine
ER: endoplasmic reticulum
GlpF: glycerol facilitator
HS-AFM: high-speed atomic force microscopy
PIP: plasma membrane intrinsic protein
POPE: 1-palmitoyl-2-oleoyl-*sn*-glycero-3-phosphoethanolamine
POPG: 1-palmitoyl-2-oleoyl-*sn*-glycero-3-phosphoglycerol
POPS: 1-hexadecanoyl-2-(9Z-octadecenoyl)-*sn*-glycero-3-phosphoserine
PSM: (*N*-palmitoyl-d-sphingomyelin)
PDB: Protein Data Bank.

## References

[1] Popot, J.L. and Engelman, D.M. (1990) Membrane protein folding and oligomerization: the two-stage model. Biochemistry 29, 4031–4037 10.1021/bi00469a001 1694455

[2] Lemmon, M.A. and Engelman, D.M. (1994) Specificity and promiscuity in membrane helix interactions. FEBS Lett. 346, 17–20 10.1016/0014-5793(94)00467-6 8206151

[3] White, S.H. and von Heijne, G. (2005) Transmembrane helices before, during, and after insertion. Curr. Opin. Struct. Biol. 15, 378–386 10.1016/j.sbi.2005.07.004 16043344

[4] Hegde, R.S. and Keenan, R.J. (2024) A unifying model for membrane protein biogenesis. Nat. Struct. Mol. Biol. 31, 1009–1017 10.1038/s41594-024-01296-5 38811793 PMC7616256

[5] Shimamoto, K., Fujikawa, K., Osawa, T., Mori, S., Nomura, K. and Nishiyama, K.I. (2024) Key contributions of a glycolipid to membrane protein integration. Proc. Jpn. Acad. Ser. B Phys. Biol. Sci. 100, 387–413 10.2183/pjab.100.026 PMC11413397 39085064

[6] Yun, S.D., Scott, E., Chang, J.Y., Bahramimoghaddam, H., Lynn, M., Lantz, C. et al. (2024) Capturing RAS oligomerization on a membrane. Proc. Natl Acad. Sci. U.S.A. 121, e2405986121 10.1073/pnas.24059861 39145928 PMC11348296

[7] Dingjan, T. and Futerman, A.H. (2021) The fine-tuning of cell membrane lipid bilayers accentuates their compositional complexity. Bioessays 43, e2100021 10.1002/bies.202100021 33656770

[8] Buck, T.M., Wagner, J., Grund, S. and Skach, W.R. (2007) A novel tripartite motif involved in aquaporin topogenesis, monomer folding and tetramerization. Nat. Struct. Mol. Biol. 14, 762–769 10.1038/nsmb1275 17632520

[9] Orlikowska, M., Wyciszkiewicz, A., Węgrzyn, K., Mehringer, J., de Souza Paiva, D. and Jurczak, P. (2024) Methods for monitoring protein-membrane binding. Comparison based on the interactions between amyloidogenic protein human cystatin C and phospholipid liposomes. Int. J. Biol. Macromol. 278, 134889 10.1016/j.ijbiomac.2024.134889 39168225

[10] Nogales, E. and Mahamid, J. (2024) Bridging structural and cell biology with cryo-electron microscopy. Nature 628, 47–56 10.1038/s41586-024-07198-2 38570716 PMC11211576

[11] Cruz-León, S., Majtner, T., Hoffmann, P.C., Kreysing, J.P., Kehl, S., Tuijtel, M.W. et al. (2024) High-confidence 3D template matching for cryo-electron tomography. Nat. Commun. 15, 3992 10.1038/s41467-024-47839-8 38734767 PMC11088655

[12] Gemmer, M., Chaillet, M.L., van Loenhout, J., Cuevas Arenas, R., Vismpas, D., Gröllers-Mulderij, M. et al. (2023) Visualization of translation and protein biogenesis at the ER membrane. Nature 614, 160–167 10.1038/s41586-022-05638-5 36697828 PMC9892003

[13] Ali, A.A. and You, M. (2024) DNA-modulated dimerization and oligomerization of cell membrane receptors. Chem. Commun. 60, 10265–10279 10.1039/d4cc03077j PMC11415102 39190295

[14] Šneiderienė, G., Czekalska, M.A., Xu, C.K., Jayaram, A.K., Krainer, G., Arter, W.E. et al. (2024) α-Synuclein oligomers displace monomeric α-synuclein from lipid membranes. ACS Nano 18, 17469–17482 10.1021/acsnano.3c10889 38916260 PMC11238581

[15] Kojima, M., Abe, S., Furuta, T., Hirata, K., Yao, X., Kobayashi, A. et al. (2024) High-throughput structure determination of an intrinsically disordered protein using cell-free protein crystallization. Proc. Natl. Acad. Sci. U.S.A. 121, e2322452121 10.1073/pnas.2322452121 38861600 PMC11194560

[16] Agyemang, E., Gonneville, A.N., Tiruvadi-Krishnan, S. and Lamichhane, R. (2024) Exploring GPCR conformational dynamics using single-molecule fluorescence. Methods 226, 35–48 10.1016/j.ymeth.2024.03.011 38604413 PMC11098685

[17] Singh, P.K., Kim, S. and Smith, A.W. (2024) HER4 is a high-affinity dimerization partner for all EGFR/HER/ErbB family proteins. Protein Sci. 10, e5171 10.1002/pro.5171 PMC11401057 39276020

[18] Luo, P., Zuo, X., Bu, Y., Qian, H., Xu, C., Niu, S. et al. (2024) The cytoskeleton controls the dynamics of plasma membrane proteins and facilitates their endocytosis in plants. Plant Physiol. 30, kiae403 10.1093/plphys/kiae403 39077775

[19] Bryant, S.J., Garvey, C.J., Darwish, T.A., Georgii, R. and Bryant, G. (2024) Molecular interactions with bilayer membrane stacks using neutron and X-ray diffraction. Adv. Colloid Interface Sci. 326, 103134 10.1016/j.cis.2024.103134 38518550

[20] Pabst, G. and Keller, S. (2024) Exploring membrane asymmetry and its effects on membrane proteins. Trends Biochem. Sci. 49, 333–345 10.1016/j.tibs.2024.01.007 38355393

[21] MacKerell, A.D., Bashford, D., Bellott, M., Dunbrack, R.L., Evanseck, J.D., Field, M.J. et al. (1998) All-atom empirical potential for molecular modeling and dynamics studies of proteins. J. Phys. Chem. B 102, 3586–3616 10.1021/jp973084f 24889800

[22] Shahane, G., Ding, W., Palaiokostas, M. and Orsi, M. (2019) Physical properties of model biological lipid bilayers: insights from all-atom molecular dynamics simulations. J. Mol. Model. 25, 76 10.1007/s00894-019-3964-0 30806797

[23] Drabik, D., Chodaczek, G., Kraszewski, S. and Langner, M. (2020) Mechanical properties determination of DMPC, DPPC, DSPC, and HSPC solid-ordered bilayers. Langmuir 36, 3826–3835 10.1021/acs.langmuir.0c00475 32176506 PMC7467745

[24] Korshunova, K., Kiuru, J., Liekkinen, J., Enkavi, G., Vattulainen, I. and Bruininks, B.M.H. (2024) Martini 3 OliGo̅mers: a scalable approach for multimers and fibrils in GROMACS. J. Chem. Theory Comput. 20, 7635–7645 10.1021/acs.jctc.4c00677 39189419 PMC11391574

[25] Burley, S.K., Berman, H.M., Bhikadiya, C., Bi, C., Chen, L., Di, L. et al. (2019) Protein Data Bank: the single global archive for 3D macromolecular structure data. Nucleic Acids Res. 47, D520–D528 10.1093/nar/gky949 30357364 PMC6324056

[26] Taylor, R. and Wood, P.A. (2019) A million crystal structures: the whole is greater than the sum of its parts. Chem. Rev. 119, 9427–9477 10.1021/acs.chemrev.9b00155 31244003

[27] Jumper, J., Evans, R., Pritzel, A., Green, T., Figurnov, M., Ronneberger, O. et al. (2021) Highly accurate protein structure prediction with AlphaFold. Nature 596, 583–589 10.1038/s41586-021-03819-2 34265844 PMC8371605

[28] Abramson, J., Adler, J., Dunger, J., Evans, R., Green, T., Pritzel, A. et al. (2024) Accurate structure prediction of biomolecular interactions with AlphaFold 3. Nature 630, 493–500 10.1038/s41586-024-07487-wi 38718835 PMC11168924

[29] Baek, M., DiMaio, F., Anishchenko, I., Dauparas, J., Ovchinnikov, S., Lee, G.R. et al. (2021) Accurate prediction of protein structures and interactions using a three-track neural network. Science. 373, 871–876 10.1126/science.abj8754 34282049 PMC7612213

[30] Krishna, R., Wang, J., Ahern, W., Sturmfels, P., Venkatesh, P., Kalvet, I. et al. (2024) Generalized biomolecular modeling and design with RoseTTAFold All-Atom. Science 384, eadl2528 10.1126/science.adl252838452047

[31] Hekkelman, M.L., de Vries, I., Joosten, R.P. and Perrakis, A. (2023) AlphaFill: enriching AlphaFold models with ligands and cofactors. Nat. Methods 20, 205–213 10.1038/s41592-022-01685-y 36424442 PMC9911346

[32] Evans, R., O'Neill, M., Pritzel, A., Antropova, N., Senior, A., Green, T. et al. (2022) Protein complex prediction with AlphaFold-Multimer. bioRxiv https://doi.org/10.1101/2021.10.04.463034

[33] Cianfrocco, M.A., Wong-Barnum, M., Youn, C., Wagner, R. and Leschziner, A. (2017) COSMIC2: A science gateway for cryo-electron microscopy structure determination. In: Practice and Experience in Advanced Research Computing 2017: Sustainability, Success and Impact 22, 1–5 10.1145/3093338.3093390

[34] Klingenberg, M. (1981) Membrane protein oligomeric structure and transport function. Nature 290, 449–454 10.1038/290449a0 6261141

[35] Cymer, F. and Schneider, D. (2012) Oligomerization of polytopic α-helical membrane proteins: causes and consequences. Biol. Chem. 393, 1215–1230 10.1515/hsz-2012-0231 23096346

[36] Jiang, Y., Thienpont, B., Sapuru, V., Hite, R.K., Dittman, J.S., Sturgis, J.N. et al. (2022) Membrane-mediated protein interactions drive membrane protein organization. Nat. Commun. 13, 7373 10.1038/s41467-022-35202-8 36450733 PMC9712761

[37] Langosch, D., Herrmann, J.R., Unterreitmeier, S. and Fuchs, A. (2011) Helix-helix interaction patterns in membrane proteins, In Structural Bioinformatics of Membrane Proteins (Frishman, D., ed.), pp. 165–186. Springer, Vienna 10.1007/978-3-7091-0045-5_10

[38] von Heijne, G. (1989) Control of topology and mode of assembly of a polytopic membrane protein by positively charged residues. Nature 341, 456–458 10.1038/341456a0 2677744

[39] Ni, C. and Hong, M. (2024) Oligomerization of drug transporters: forms, functions, and mechanisms. Acta Pharm. Sin. B. 14, 1924–1938 10.1016/j.apsb.2024.01.007 38799641 PMC11119549

[40] Ridder, A.N., van de Hoef, W., Stam, J., Kuhn, A., de Kruijff, B. and Killian, J.A. (2002) Importance of hydrophobic matching for spontaneous insertion of a single-spanning membrane protein. Biochemistry 41, 4946–4952 10.1021/bi0158674 11939790

[41] Gössweiner-Mohr, N., Siligan, C., Pluhackova, K., Umlandt, L., Koefler, S., Trajkovska, N. et al. (2022) The hidden intricacies of aquaporins: remarkable details in a common structural scaffold. Small 18, e2202056 10.1002/smll.202202056 35802902

[42] Jayaraman, K., Das, A.K., Luethi, D., Szöllősi, D., Schütz, G., Reith, M.E.A. et al. (2021) SLC6 transporter oligomerization. J. Neurochem. 157, 919–929 10.1111/jnc.15145 32767560 PMC8247324

[43] Muth, L.T. and Van Bogaert, I.N.A. (2024) Let it stick: strategies and applications for intracellular plasma membrane targeting of proteins in *Saccharomyces cerevisiae*. Yeast 41, 315–329 10.1016/10.1002/yea.3933 38444057

[44] Jensen, D. and Schekman, R. (2011) COPII-mediated vesicle formation at a glance. J. Cell Sci. 124, 1–4 10.1242/jcs.069773 21172817

[45] Sitte, H.H., Farhan, H. and Javitch, J.A. (2004) Sodium-dependent neurotransmitter transporters: oligomerization as a determinant of transporter function and trafficking. Mol. Interv. 4, 38–47 10.1124/mi.4.1.38 14993475

[46] Vismpas, D. and Förster, F. (2024) RAMPing up knowledge of the translocon. Elife 13, e98548 10.7554/eLife.98548 38787756 PMC11126307

[47] Yang, Y., Valencia, L.A., Lu, C.H., Nakamoto, M.L., Tsai, C.T., Liu, C. et al. (2024) Plasma membrane curvature regulates the formation of contacts with the endoplasmic reticulum. Nat. Cell Biol. 26, 1878–1891 10.1038/s41556-024-01511-x 39289582 PMC11567891

[48] Corin, K. and Bowie, J.U. (2022) How physical forces drive the process of helical membrane protein folding. EMBO Rep. 23, e53025 10.15252/embr.202153025 35133709 PMC8892262

[49] Cymer, F. and Schneider, D. (2010) A single glutamate residue controls the oligomerization, function, and stability of the aquaglyceroporin GlpF. Biochemistry 49, 279–286 10.1021/bi901660t 20000688

[50] Lagrée, V., Froger, A., Deschamps, S., Pellerin, I., Delamarche, C., Bonnec, G. et al. (1998) Oligomerization state of water channels and glycerol facilitators. Involvement of loop E. J. Biol. Chem. 273, 33949–33953 10.1074/jbc.273.51.33949 9852047

[51] Bron, P., Lagrée, V., Froger, A., Rolland, J.P., Hubert, J.F., Delamarche, C. et al. (1999) Oligomerization state of MIP proteins expressed in *Xenopus* oocytes as revealed by freeze-fracture electron-microscopy analysis. J. Struct. Biol. 128, 287–296 10.1006/jsbi.1999.4196 10633068

[52] Kamsteeg, E.J., Wormhoudt, T.A., Rijss, J.P., van Os, C.H. and Deen, P.M. (1999) An impaired routing of wild-type aquaporin-2 after tetramerization with an aquaporin-2 mutant explains dominant nephrogenic diabetes insipidus. EMBO J. 18, 2394–2400 10.1093/emboj/18.9.2394 10228154 PMC1171322

[53] Fetter, K., Van Wilder, V., Moshelion, M. and Chaumont, F. (2004) Interactions between plasma membrane aquaporins modulate their water channel activity. Plant Cell 16, 215–228 10.1105/tpc.017194 14671024 PMC301406

[54] Yaneff, A., Sigaut, L., Marquez, M., Alleva, K., Pietrasanta, L.I. and Amodeo, G. (2014) Heteromerization of PIP aquaporins affects their intrinsic permeability. Proc. Natl. Acad. Sci. U.S.A. 111, 231–236 10.1073/pnas.1316537111 24367080 PMC3890845

[55] Chevalier, A.S. and Chaumont, F. (2015) Trafficking of plant plasma membrane aquaporins: multiple regulation levels and complex sorting signals. Plant Cell Physiol. 56, 819–829 10.1093/pcp/pcu203 25520405 PMC7107115

[56] Bienert, M.D., Diehn, T.A., Richet, N., Chaumont, F. and Bienert, G.P. (2018) Heterotetramerization of plant PIP1 and PIP2 aquaporins is an evolutionary ancient feature to guide PIP1 plasma membrane localization and function. Front. Plant Sci. 26, 382 10.3389/fpls.2018.00382 PMC5879115 29632543

[57] Vajpai, M., Mukherjee, M. and Sankararamakrishnan, R. (2018) Cooperativity in Plant Plasma Membrane Intrinsic Proteins (PIPs): mechanism of increased water transport in maize PIP1 channels in hetero-tetramers. Sci. Rep. 8, 12055 10.1038/s41598-018-30257-4 30104609 PMC6089885

[58] Hrmova, M. and Gilliham, M. (2018) Plants fighting back: to transport or not to transport, this is a structural question. Curr. Opin. Plant Biol. 46, 68–76 10.1016/j.pbi.2018.07.006 30138844

[59] Matsumoto, T., Lian, H.L., Su, W.A., Tanaka, D., Liu, C.W., Iwasaki, I. et al. (2009) Role of the aquaporin PIP1 subfamily in the chilling tolerance of rice. Plant Cell Physiol. 50, 216–229 10.1093/pcp/pcn190 19098326

[60] Jozefkowicz, C., Rosi, P., Sigaut, L., Soto, G., Pietrasanta, L.I., Amodeo, G. et al. (2013) Loop A is critical for the functional interaction of two *Beta vulgaris* PIP aquaporins. PLos One 8, e57993 10.1371/journal.pone.0057993 23483963 PMC3587573

[61] Jozefkowicz, C., Sigaut, L., Scochera, F., Soto, G., Ayub, N., Pietrasanta, L.I. et al. (2016) PIP water transport and its pH dependence are regulated by tetramer stoichiometry. Biophys. J. 110, 1312–1321 10.1016/j.bpj.2016.01.026 27028641 PMC4816683

[62] Törnroth-Horsefield, S., Wang, Y., Hedfalk, K., Johanson, U., Karlsson, M., Tajkhorshid, E. et al. (2006) Structural mechanism of plant aquaporin gating. Nature 439, 688–694 10.1038/nature04316 16340961

[63] Berny, M.C., Gilis, D., Rooman, M. and Chaumont, F. (2016) Single mutations in the transmembrane domains of maize plasma membrane aquaporins affect the activity of monomers within a heterotetramer. Mol. Plant 9, 986–1003 10.1016/j.molp.2016.04.006 27109604

[64] Jiang, J., Daniels, B.V. and Fu, D. (2006) Crystal structure of AqpZ tetramer reveals two distinct Arg-189 conformations associated with water permeation through the narrowest constriction of the water-conducting channel. J. Biol. Chem. 281, 454–460 10.1074/jbc.M508926200 16239219

[65] Horsefield, R., Nordén, K., Fellert, M., Backmark, A., Törnroth-Horsefield, S., Terwisscha van Scheltinga, A.C. et al. (2008) High-resolution x-ray structure of human aquaporin 5. Proc. Natl. Acad. Sci. U.S.A. 105, 13327–12332 10.1073/pnas.0801466105 18768791 PMC2533189

[66] Pei, J., Kim, B.-H. and Grishin, N.V. (2008) PROMALS3D: a tool for multiple protein sequence and structure alignments. Nucleic Acids Res. 36, 2295–2300 10.1093/nar/gkn072 18287115 PMC2367709

[67] Krissinel, E. and Henrick, K. (2007) Inference of macromolecular assemblies from crystalline state. J. Mol. Biol. 372, 774–797 10.1016/j.jmb.2007.05.022 17681537

[68] Jo, S., Kim, T., Iyer, V.G. and Im, W. (2008) CHARMM-GUI: a web-based graphical user interface for CHARMM. J. Comput. Chem. 29, 1859–1865 10.1002/jcc.20945 18351591

[69] Klauda, J.B., Venable, R.M., Alfredo, F.J., O'Connor, J.W., Tobias, D.J., Mondragon-Ramirez, C. et al. (2010) Update of the CHARMM all-atom additive force field for lipids: validation on six lipid types. J. Phys. Chem. B 114, 7830–7843 10.1021/jp101759q 20496934 PMC2922408

[70] Krieger, E., Joo, K., Lee, J., Lee, J., Raman, S., Thompson, J. et al. (2009) Improving physical realism, stereochemistry, and side-chain accuracy in homology modeling: four approaches that performed well in CASP8. Proteins 77, 114–122 10.1002/prot.22570 19768677 PMC2922016

[71] Krieger, E., Darden, T., Nabuurs, S.B., Finkelstein, A. and Vriend, G. (2004) Making optimal use of empirical energy functions: force-field parameterization in crystal space. Proteins 57, 678–683 10.1002/prot.20251 15390263

[72] Klein, N., Hellmann, N. and Schneider, D. (2015) Anionic lipids modulate the activity of the aquaglyceroporin GlpF. Biophys. J. 109, 722–731 10.1016/j.bpj.2015.06.063 26287624 PMC4547167

[73] Schmidt, V., Sidore, M., Bechara, C., Duneau, J.P. and Sturgis, J.N. (2019) The lipid environment of *Escherichia coli* aquaporin Z. Biochim. Biophys. Acta Biomembr. 1861, 431–440 10.1016/j.bbamem.2018.10.017 30414848

[74] Briones, R., Aponte-Santamaría, C. and de Groot, B.L. (2017) Localization and ordering of lipids around aquaporin-0: protein and lipid mobility effects. Front. Physiol. 8, 124 10.3389/fphys.2017.00124 28303107 PMC5332469

[75] Gonen, T., Cheng, Y., Sliz, P., Hiroaki, Y., Fujiyoshi, Y., Harrison, S.C. et al. (2005) Lipid-protein interactions in double-layered two-dimensional AQP0 crystals. Nature 438, 633–638 10.1038/nature04321 16319884 PMC1350984

[76] Chiu, P.L., Orjuela, J.D., de Groot, B.L., Aponte Santamaría, C. and Walz, T. (2024) Structure and dynamics of cholesterol-mediated aquaporin-0 arrays and implications for lipid rafts. Elife 12, RP90851 10.7554/eLife.90851 39222068 PMC11368405

